# Using EEG Alpha States to Understand Learning During Alpha Neurofeedback Training for Chronic Pain

**DOI:** 10.3389/fnins.2020.620666

**Published:** 2021-02-22

**Authors:** Kajal Patel, James Henshaw, Heather Sutherland, Jason R. Taylor, Alexander J. Casson, Karen Lopez-Diaz, Christopher A. Brown, Anthony K. P. Jones, Manoj Sivan, Nelson J. Trujillo-Barreto

**Affiliations:** ^1^School of Medicine, University of Manchester, Manchester, United Kingdom; ^2^Division of Neuroscience and Experimental Psychology, University of Manchester, Manchester, United Kingdom; ^3^Department of Electrical and Electronic Engineering, University of Manchester, Manchester, United Kingdom; ^4^Department of Psychological Sciences, University of Liverpool, Liverpool, United Kingdom; ^5^Academic Department of Rehabilitation Medicine, University of Leeds, Leeds, United Kingdom

**Keywords:** alpha states, alpha rhythm, neurofeedback, EEG biofeedback, chronic pain

## Abstract

**Objective:**

Alpha-neurofeedback (α-NFB) is a novel therapy which trains individuals to volitionally increase their alpha power to improve pain. Learning during NFB is commonly measured using static parameters such as mean alpha power. Considering the biphasic nature of alpha rhythm (high and low alpha), dynamic parameters describing the time spent by individuals in high alpha state and the pattern of transitioning between states might be more useful. Here, we quantify the changes during α-NFB for chronic pain in terms of dynamic changes in alpha states.

**Methods:**

Four chronic pain and four healthy participants received five NFB sessions designed to increase frontal alpha power. Changes in pain resilience were measured using visual analogue scale (VAS) during repeated cold-pressor tests (CPT). Changes in alpha state static and dynamic parameters such as fractional occupancy (time in high alpha state), dwell time (length of high alpha state) and transition probability (probability of moving from low to high alpha state) were analyzed using Friedman’s Test and correlated with changes in pain scores using Pearson’s correlation.

**Results:**

There was no significant change in mean frontal alpha power during NFB. There was a trend of an increase in fractional occupancy, mean dwell duration and transition probability of high alpha state over the five sessions in chronic pain patients only. Significant correlations were observed between change in pain scores and fractional occupancy (*r* = −0.45, *p* = 0.03), mean dwell time (*r* = -0.48, *p* = 0.04) and transition probability from a low to high state (*r* = -0.47, *p* = 0.03) in chronic pain patients but not in healthy participants.

**Conclusion:**

There is a differential effect between patients and healthy participants in terms of correlation between change in pain scores and alpha state parameters. Parameters providing a more precise description of the alpha power dynamics than the mean may help understand the therapeutic effect of neurofeedback on chronic pain.

## Introduction

Neurofeedback (NFB) is a neuromodulatory therapy which trains patients to develop volitional control over their brain activity (Patel et al., 2020). Neurofeedback systems provide patients with a real-time representation of their electroencephalogram (EEG) signals (Alkoby et al., 2018). This facilitates recognition and practice of mental strategies that allow them to achieve brain states associated with therapeutic benefit (Bagdasaryan and Le Van Quyen, 2013). NFB has been implemented in a variety of conditions ranging from anxiety, depression to chronic pain with promising results being reported by several studies (Schoenberg and David, 2014; Melo et al., 2019).

One of the areas where neurofeedback has been increasingly explored is chronic pain. Alpha power has been known to be lower in chronic pain patients compared to healthy individuals in a number of chronic pain conditions (Chang et al., 2001; Boord et al., 2008; Saithong et al., 2012; Jensen et al., 2013b; Lim et al., 2016; Nickel et al., 2017). Hence, several studies have attempted to increase the alpha power in these patient groups using neurofeedback with the aim of alleviating pain either by targeting alpha rhythm in isolation (Elbogen et al., 2019; Mayaud et al., 2019) or in combination with other rhythms like beta and theta rhythms (Jensen et al., 2013a; Hassan et al., 2015; Al-Taleb et al., 2019; Vuèkoviæ et al., 2019). Whilst most of these studies report a significant reduction in pain in these individuals following neurofeedback, very few of these studies have been able to show a direct correlation between the reduction in pain and the change in neurophysiological signal as highlighted by a recent systematic review (Patel et al., 2020). All of the neurofeedback studies conducted in the past decade have used mean alpha power to measure changes in neurophysiological signals (Jensen et al., 2013a; Hassan et al., 2015; Al-Taleb et al., 2019; Elbogen et al., 2019; Mayaud et al., 2019; Vuèkoviæ et al., 2019). This raises the question of whether the indices commonly used to gauge the success of learning truly reflect the neurophysiological changes underlying pain relief following neurofeedback.

The choice of learning index has indeed been a highly debated topic in the field of neurofeedback. Two widely used indices include mean alpha power and percentage time above a pre-determined alpha power threshold (Travis et al., 1974; Hardt and Kamiya, 1976; Lansky et al., 1979; Dempster and Vernon, 2009). Whilst some researchers believe that mean alpha power is the most sensitive index of the two (Hardt and Kamiya, 1976; Dempster and Vernon, 2009), others have argued that dynamic indices might be more informative. For instance, early work found that durations of periods of high alpha power obeyed a non-trivial asymmetrically shifted exponential distribution (Bohdaneck et al., 1978). A recent study (Ossadtchi et al., 2017) looked at changes following neurofeedback in terms of alpha spindles and reported that there was an increase only in frequency of alpha spindles with no change in the amplitude of these spindles. Whilst there are not many studies in the field of neurofeedback and chronic pain that have taken this approach of analyzing dynamic nature of alpha rhythm, the idea of bi-modal alpha amplitude states is being increasingly explored in other fields as discussed below.

It has been shown that the alpha rhythm has bi-stable dynamics, whereby the alpha power erratically jumps between high and low amplitude modes or states (Freyer et al., 2009; Roberts et al., 2015). Changes in such bi-stable dynamics following any form of intervention can be captured in a number of ways. For instance, one can measure the amount of total time that an individual stays in the high (low) alpha state, also referred to as fractional occupancy, or the chance of transitioning from one state to the other, also known as transition probabilities (Khanna et al., 2014; Quinn et al., 2018; Kottaram et al., 2019). To explore this further, some studies have also mapped out the distribution of high alpha state durations (Quinn et al., 2018). Such measures have been shown to correlate with motor and cognitive function in Parkinson’s disease (Chu et al., 2020) and schizophrenia (Khanna et al., 2015) and furthermore, cognitive manipulation of these states can be achieved through interventions (Seitzman et al., 2017). However, no studies have used these dynamic parameters of alpha rhythm to measure neurophysiological changes in chronic pain.”

It is not clear how well the currently used indices capture such complex dynamic expression of the alpha rhythm. Understanding the temporal changes in these states might give us more insight into neuronal mechanisms which underlie pain processing as higher alpha rhythm has been associated with increased resilience to pain (Jensen et al., 2013b; Lim et al., 2016; Villafaina et al., 2019). Furthermore, distribution of times that the neuronal networks dwell in each state and the pattern of transition between the states might be key to understanding how these systems process painful stimuli as well as provide insight into the mechanism by which NFB alters neuronal signaling and pain processing.

**FIGURE 1:**
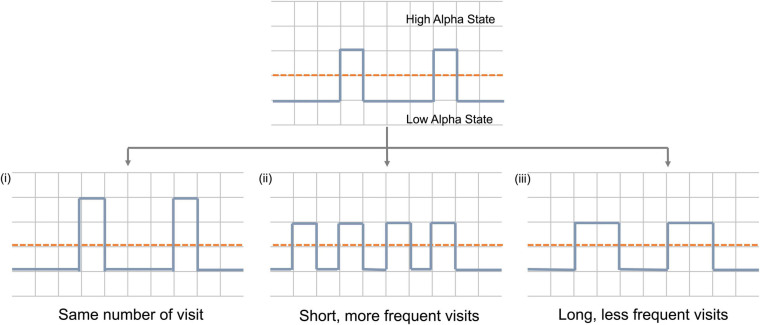
Schematic representation of mechanisms by which alpha power can increase during neurofeedback. Dashed red line represent pre-set threshold defining the states and solid blue line represents alpha power during neurofeedback. Y-axis represents time and x-axis represents alpha power.

Brain activity can be assumed to occupy one of the two alpha states over time. An increase in average alpha power can be achieved in one of three ways (or a combination of them) (Figure 1): (i). Firstly, due to an increase in the power of the high alpha state with the number of visits remaining constant; (ii) due to more frequent visits to the high alpha state and (iii) due to longer time spent in each visit to that high state.

Whilst there are many ways in which brain activity may be modulated, it is unclear which of these parameters are more sensitive to the effect of NFB training or whether a combination of them will describe individual differences better (specificity). It might be the case that it is possible to voluntarily control alpha activity only through one of these mechanisms. The sensitivity and/or specificity of these parameters may vary between chronic pain patients and healthy participants. More importantly, it is not known how changes in any one of these parameters correlate with changes in behavioral outcomes. Therefore, in order to be able to sensitively measure meaningful NFB learning, a greater understanding of the temporal dynamics of alpha power changes, their susceptibility to voluntary control and their correlation to behavioral outcomes is required.

This study attempted to understand the changes in temporal dynamics of EEG which occur during alpha NFB using a bimodal alpha states model. Brain alpha states analysis was conducted on electroencephalogram (EEG) data during five α-NFB sessions in chronic pain patients as well as healthy participants to gain an insight into differences in the way brain activity changes in these two groups during the intervention.

## Materials and Methods

### Study Design

This was an exploratory study conducted in the Human Pain Research Group laboratory at Salford Royal Hospital, United Kingdom, with the aim of testing the proof-of-concept of an alpha NFB system in training individuals to modulate their alpha activity in order to increase their resilience to pain. This study was sponsored by the University of Manchester and approved by the National NHS Research Ethics Committee (REC reference 18/NS/0102, IRAS ID 244779). Written informed consent was obtained from the participants according to the Declaration of Helsinki. This study was a registered clinical trial NCT04097522.

### Participant Recruitment

**FIGURE 2:**
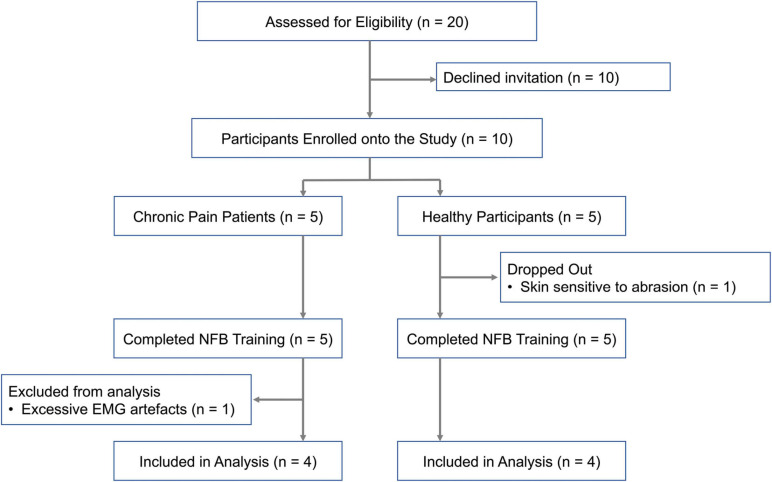
Flow diagram of participant recruitment onto each stage of the study.

Participant recruitment has been summarized in Figure 2. Ten participants (6 females, 4 males) were recruited for the study. Chronic pain conditions studied included fibromyalgia, chronic headache and lower back pain. EEG data from eight participants, four healthy participants and four chronic pain patients, who completed all five neurofeedback sessions was included in the final analysis. The patient group was heterogeneous including a range of chronic pain conditions in order to make the results widely applicable to chronic pain in general. Adults above the age of 18 years who were able to give informed consent were eligible. Exclusion criteria included concomitant psychotherapy, previous brain injury, stroke or surgery, and any brain or spinal cord implants.

### General Procedure

NFB training consisted of five sessions spread across approximately 3 weeks. The experimental protocol and conditions were same for both the groups. At the beginning and end of each session, resting state alpha power was recorded with eyes-open for 2 min. Pain resilience was also assessed at the start and end of each session before measuring resting-state alpha activity by inducing experimental pain using Cold-Pressor Test (CPT) at 10°C cold water for 3 min. The participants were asked to give a pain rating on the scale of 0 (no pain) to 10 (worst pain) using the Visual Analogue Scale (VAS) every 30 s. An average pain rating was obtained by calculating the mean of the six VAS pain ratings provided over the course of the 3 min CPT block. Change in pain scores was calculated for each session by comparing pain rating in each session with pain ratings before any NFB.

### Neurofeedback System

EEG was acquired using a 64-channel Standard BrainCap-MR with multitrodes by Brain Products (Herrsching, Germany) (actiCHamp Plus (64 channels) [Apparatus], 2019). The NFB system used a sampling frequency of 1,000 Hz and channel impedances were kept below 20 kΩ. All electrodes were referenced to channel Fz with AFz used as the ground electrode. Neurofeedback was delivered using an in-house developed MATLAB script. A 10-s sliding window was used for filtering the EEG data at the frequency band of interest which was 8–13 Hz. Power calculation and feedback were then provided on the last 2-s period of data from this 10-s sliding window.

Each session consisted of two NFB training blocks of 5 min each with a 1 min break between the blocks. Participants were provided with continuous visual feedback in the form of a dial ranging from 0 to 10, where an increase in mean alpha power recorded from frontal channels AF3 and AF4 caused the arrow on the dial to move toward 10 and vice versa. The participants were instructed to keep their needle on or close to 10 for the duration of training. The 0 and 10 corresponded to 2nd and 98th percentile of their alpha power during resting-state respectively. Feedback was provided for alpha frequency (8–13 Hz) from frontal electrodes AF3 and AF4.

### Off-Line Analysis

All the EEG pre-processing and analysis was performed using EEGLab (Delorme and Makeig, 2004) and fieldtrip (Oostenveld et al., 2011) toolboxes through a script written in MATLAB 2019a 9.6 software (Mathworks Inc., United States) (Natick MTMI. MATLAB, 2018). The raw signal data from AF3 and AF4 were down sampled to 250 Hz and segmented into 1 s non-overlapping epochs. The EEG acquired was first cleaned by visual inspection to remove epochs with high amplitude technical artifacts. No more than 10% of the total epochs were discarded during process. Independent Component Analysis (ICA) was then used to remove components associated with eye blinks, eye movements and muscle movement using SASICA plugin. On average 3–5 components were removed. Frequency analysis was performed using Fourier Transformation to obtain the average alpha power in the frequency range 8–13 Hz for each 1 s epoch of data gathered during resting state and NFB block.

### EEG Brain Alpha States Parameters

The dynamics of alpha power fluctuations were first characterized using a symbolic dynamic method. Each 1s epoch was labeled as “1,” if the mean power was higher than a certain pre-defined threshold (high alpha state) or “0” if the power was lower than the threshold. The threshold was computed individually for each participant and defined as a percentage of the maximum alpha power during the resting-state eyes-open EEG from the first session. To assess the sensitivity of the analysis to threshold choice, three different threshold values were analyzed, 30, 50, and 70%. The maximum alpha power was defined as 1.5 × interquartile range for each individual. This was done in order to prevent random high-amplitude fluctuations in alpha power from being used to set a threshold.

After symbolization, the following alpha state parameters were calculated based on the state sequences obtained:

• Fractional Occupancy: Defined as the fraction of all epochs occupied by high alpha state.
• Dwell Time (duration) Distribution: Defined as the frequency of dwell times of each state during neurofeedback. Dwell time of the high (low) state is computed as the counts of contiguous epochs where the alpha power was successively in the high (low) state before transitioning to the low (high) state in each state visit. The distribution was then plotted as a violin chart and described in terms of mean, median, mode, variance and tail weights for each plot.
• Transition Probability: Defined as the likelihood (probability) of transitioning from one state to another. This was estimated by assuming an observable Markov Process to explain the state sequences. This was achieved by using the hesitate() function on MATLAB [which is based on a Hidden Markov Model (Quinn et al., 2018)], and forcing the states’ emission probability to be the identity matrix.

Analysis based on these dynamic parameters was contrasted with more traditional (static) evaluation based on the normalized and log-transformed mean alpha power in each NFB session. Each parameter from a single neurofeedback session was then correlated with change in pain scores reported by the participant in that particular session.

### Statistical Analysis

Statistical analysis was performed using IBM SPSS Statistics version 25 (IBM Corp, 2017). Each alpha state parameter was analyzed using a Friedman Test in order to analyze changes in repeated recordings of alpha power parameters over the five neurofeedback sessions. Correlation between these parameters and behavioral outcomes were tested using Pearson’s correlation. Use of parametric tests was possible due to availability of more datapoints and a normal distribution of these points. Generally, statistical significance was accepted with a *p*-value of less than 0.05 for all the tests.

## Results

### Changes in Mean Alpha Power

**FIGURE 3:**
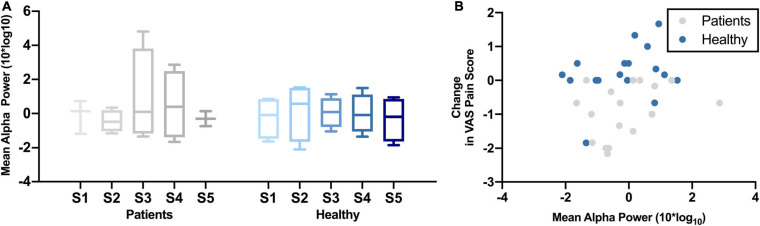
**(A)** Normalized Mean Alpha Power over five neurofeedback sessions in chronic pain patients and healthy participants. NFB sessions are denoted by S1–S5. **(B)** Change in mean alpha power during a neurofeedback session and change in VAS pain ratings.

Figure 3A shows the change in mean alpha power over the five NFB sessions in chronic pain patients and healthy participants. There was no significant change in mean frontal alpha power over the five NFB sessions in patients [χ^2^(4) = 0.80, *p* = 0.94] or healthy participants [χ^2^(4) = 1.40, *p* = 0.84]. The correlation between mean alpha power and change in pain scores during cold-pressor test in that session were as follows: chronic pain patients *r* = 0.32, *p* = 0.21; Healthy participants *r* = 0.44, *p* = 0.05 (Figure 3B).

### EEG Alpha State Temporal Characteristics

**FIGURE 4:**
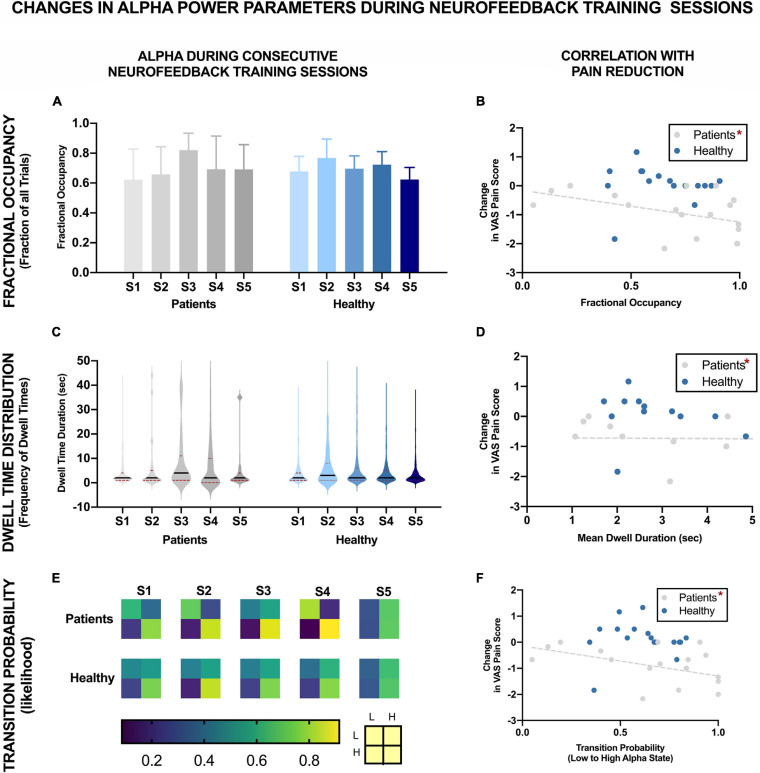
EEG Brain alpha states characteristics over five NFB sessions in chronic pain patients and healthy participants and their correlation with reduction in VAS pain ratings. The NFB sessions are denoted by S1–S5. Error bars show standard error. L refers to low alpha state and H refers to high alpha state. *Statistically significance of *p* < 0.05.

Figure 4 shows the changes in different alpha state parameters over five NFB sessions in chronic pain patients and healthy participants. Descriptive statistics of these parameters are provided in Table 1.

**TABLE 1:** Descriptive statistics of EEG alpha state parameters over five NFB sessions in chronic pain patients and healthy participants.

	**Chronic pain patients**					**Healthy participants**				
	**S1**	**S2**	**S3**	**S4**	**S5**	**S1**	**S2**	**S3**	**S4**	**S5**
**Mean amplitude**										
Mean (*SD*)	-0.10 (0.98)	-0.44 (0.64)	0.92 (2.75)	0.50 (2.01)	−3.0 (0.43)	−0.24 (1.27)	0.14 (1.71)	0.07 (0.89)	-0.01 (1.17)	-0.32 (1.32)
**Fractional occupancy**										
Mean (*SD*)	0.62 (0.35)	0.66 (0.37)	0.82 (0.23)	0.69 (0.45)	0.69 (0.29)	0.68 (0.20)	0.77 (0.25)	0.70 (0.17)	0.72 (0.17)	0.62 (0.16)
**Dwell times**										
Mean	4.13	6.45	9.09	6.82	5.99	3.86	6.09	4.98	4.07	3.27
Median	2.00	2.00	4.00	2.00	2.00	2.00	3.00	2.00	2.00	2.00
Mode	2.00	1.00	2.00	2.00	1.00	1.00	2.00	1.00	1.00	1.00
Variance	133%	168%	129%	145%	174%	133%	115%	137%	122%	121%
Tail wgt.	1.96	2.55	1.84	3.96	2.04	1.98	2.21	2.21	2.18	2.54
**Transition probability**										
Low > Low	0.63	0.72	0.43	0.81	0.70	0.47	0.45	0.43	0.38	0.34
Low > High	0.37	0.28	0.57	0.19	0.30	0.53	0.55	0.57	0.62	0.66
High > High	0.78	0.84	0.88	0.91	0.71	0.75	0.84	0.75	0.76	0.69
High > Low	0.22	0.16	0.12	0.09	0.29	0.25	0.16	0.25	0.24	0.31

*Threshold for transition to high alpha state was set at 30% of maximum alpha power. The NFB sessions are denoted by S1–S5.*

#### Fractional Occupancy

There was no significant change in fractional occupancy over the five NFB sessions in patients [χ^2^(4) = 1.97, *p* = 0.74] or healthy [χ^2^(4) = 4.40, *p* = 0.36] participants. However, a gradual increase in mean fractional occupancy was seen in chronic pain patients with each subsequent session until the fourth session where there was a drop compared to the third session, nevertheless, the fractional occupancy in the fourth and fifth session remained higher than the first session (Figure 4A).

#### Dwell Time Distribution

Dwell times followed a heavy tail distribution whereby most visits to the high alpha state were of short duration (Figure 4C). The frequency of visits decreased as the dwell duration increase. There was no significant change in mean dwell time over the five NFB sessions in patients [χ^2^(4) = 2.13, *p* = 0.71] or healthy participants [χ^2^(4) = 3.00, *p* = 0.56]. Although, the results were not statistically significant, there were some important trends in data over sessions. Over the course of the NFB training, there was an increase in the heaviness of the tail in chronic pain patients as shown by the increasing thickness of tails in Figure 4C until session five. There were more visits with longer dwell times over the course of the neurofeedback training. There was also a slight increase in the length of the tail until session five. In contrast, the distribution of dwell times in healthy participants did not show consistent change over the five sessions.

#### Transition Probability

Figure 4E shows a heat-map demonstrating the probabilities of transitioning from low to high alpha state and vice versa as well as the probabilities of remaining in a low or high state during each NFB session. Statistical analysis performed on transition probability from low to high alpha state showed that there was no significant change in probability of transitioning from low to high alpha state over the five NFB sessions in patients [χ^2^(4) = 2.57, *p* = 0.63] or healthy participants [χ^2^(4) = 6.20, *p* = 0.18].

However, there were some overall trends which emerged when considering all of the transition probabilities. During the first session, compared to chronic pain patients, healthy participants had a slightly higher probability of transitioning from low to high alpha state, lower probability of moving from high to low alpha state, were more likely to remain in high alpha state and less likely to remain in the low alpha state. Over the course of training, in the chronic pain group, there was no trend in the probability of transitioning from low to high alpha state. There was a decrease in the probability of transitioning from high to low state. There was an increase in the probability of patients remaining in high alpha state which increased with each session until the last session.

**FIGURE 5:**
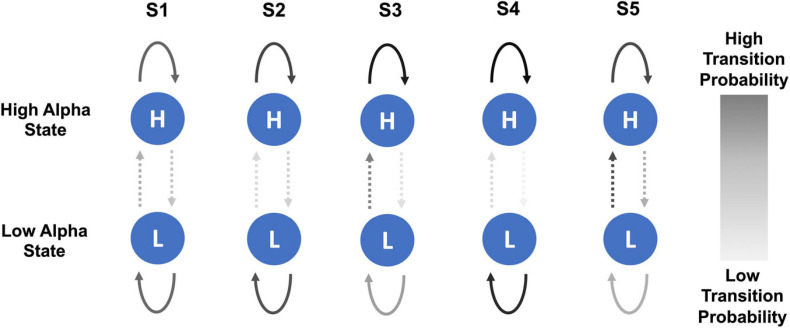
Schematic representation of changes in probability of transitioning from low to high alpha state and vice versa over the course of five NFB sessions in chronic pain patients. The NFB sessions are denoted by S1–S5.

In the healthy participant group, there was a trend of a small increase in the probability of transitioning from low to high alpha state over the five sessions but no steady change in the probability of transitioning from high to low state. Figure 5 shows a schematic representation of changes in the probability of transitioning from low to high alpha state over five neurofeedback sessions in chronic pain patients.

#### Correlation With Reduction in VAS Pain Ratings

Figures 4B,D,F show the correlation of each of the EEG alpha state parameter with reduction in VAS pain scores. None of the parameters were significantly correlated with change in pain scores in healthy participants including: Fractional Occupancy (*r* = 0.19, *p* = 0.20), Mean Dwell Duration (*r* = -0.22, *p* = 0.34), Transition Probability from low to high alpha state (*r* = 0.03, *p* = 0.46). In the chronic pain group, there was a significant negative correlation between change in pain scores and Fractional Occupancy (*r* = -0.45, *p* = 0.03), Mean Dwell Times (*r* = -0.48, *p* = 0.04) as well as Transition Probability from low to high alpha state (*r* = -0.47, *p* = 0.03).

### Sensitivity to Thresholds

#### Sensitivity of Parameters to Threshold

**FIGURE 6:**
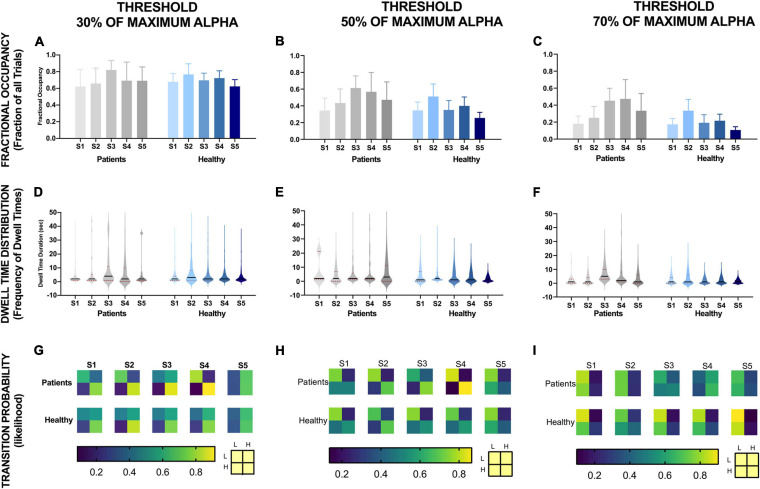
EEG alpha state parameters over five NFB sessions in chronic pain patients and healthy participants for different thresholds. NFB sessions are denoted by S1–S5.

Figure 6 shows how changes in different alpha state parameters differ for different thresholds.

Fractional occupancy of chronic pain participants during the first session was similar to healthy participants for each threshold (Figures 6A–C). Although statistically non-significant, for all thresholds, chronic pain participants then showed a trend of an increase in fractional occupancy over the first three sessions, with a drop in the last two sessions. Nevertheless, the fractional occupancy in the last two sessions remained above that observed in the first session. Furthermore, the slope of change in fractional occupancy was steeper at higher thresholds (Figure 6C) compared to lower thresholds (Figure 6A). There was no consistent change in fractional occupancy in healthy participants over sessions.

Dwell time distribution of chronic pain patients was also similar to healthy participants across all thresholds during the first session (Figures 6D–F). However, across all thresholds, this distribution did not change much over sessions for healthy participants. However, the heaviness and the length of the tail increased over sessions in chronic pain patients. The increase in heaviness and length of tail was more prominent at higher thresholds. However, these changes in mean amplitude over sessions was not statistically significant.

The transition probability matrices of chronic pain patients were similar to healthy participants at the beginning of training across all thresholds. Overall, across all thresholds, there was a general trend of an increase in the probability of transitioning from low to high alpha state and decrease in probability of transitioning from high alpha to low alpha state over sessions in both chronic pain patients and healthy participants across all thresholds except for the last session. However, the change in probability was greater for lower thresholds compared to high thresholds and greater for chronic pain patients compared to healthy participants.

#### Sensitivity of Correlation to Threshold

**FIGURE 7:**
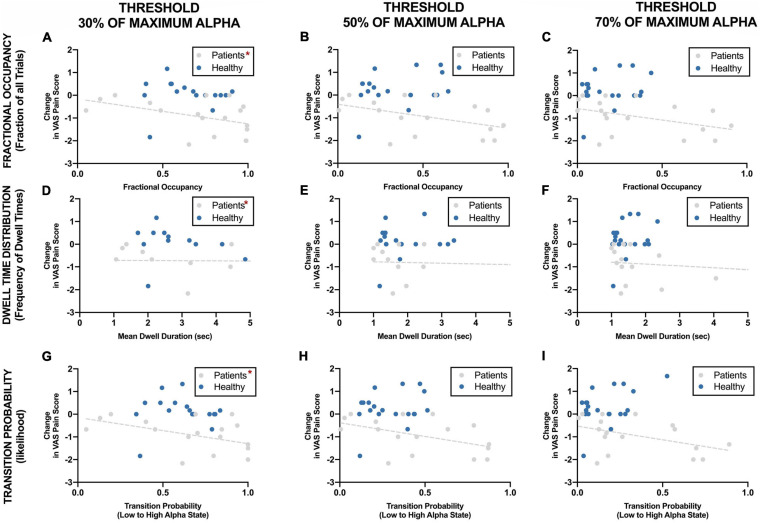
Correlation between EEG alpha state parameters using different thresholds and reduction in VAS pain ratings during neurofeedback sessions. *Statistically significance of *p* < 0.05.

Figure 7 shows the correlation of different parameters at different threshold with reduction in VAS pain scores. Change in pain scores was significantly correlated with fractional occupancy, mean dwell time and transition probability when the threshold was set at 30% of maximum alpha power as discussed above. These correlations were significant only in the chronic pain patients and not in the healthy participants. These correlations were not significant when the thresholds of 50% and 70% of maximum resting-state alpha power were used. Interestingly, there was a cluster of datapoints with fractional occupancy and transition probability much higher than the rest of the chronic pain patient group, which could potentially affect the results of the statistical tests.

#### Baseline EEG Alpha Parameters

Supplementary Figure 1 shows EEG Alpha state parameters at baseline before any neurofeedback was delivered in the two groups. There was no statistically significant difference in the mean alpha power, fractional occupancy, dwell time distribution or transition probability between healthy participants and chronic pain patients at rest. Although chronic pain patients had a longer and heavier tail for dwell time distribution, the difference in the mean dwell time was not significant between the groups.

## Discussion

In this study, we found that over five alpha neurofeedback sessions, there was no increase in mean alpha power in patients, however, there was a trend of increase in other alpha state parameters such as fractional occupancy, dwell time distribution and transition probability. Our results suggest that with neurofeedback training, participants were more likely to make transitions from a low alpha state to a high alpha state and once in the high alpha state, they were more likely to remain in that state before reverting back to low alpha state. Such trends were consistent only in chronic pain patients and not in healthy participants. This is the first study to our knowledge to have compared mean alpha power and alpha state parameters following NFB for pain. We propose an EEG alpha states-based approach to analyzing learning following neurofeedback and provide interesting insight into changes in neurophysiological signals that occur with neurofeedback learning as well as link this to possible mechanisms that may underlie the therapeutic benefit of this neuromodulatory technique.

Negative correlation of alpha state parameters with changes in VAS pain rating during cold-pressor tests was statistically significant for fractional occupancy, mean dwell time and transition probability, meaning that we saw that the greater the fractional occupancy, the longer the high alpha state visit and the higher the transition probability from low to high state, the greater the reduction in pain. Furthermore, these correlations were sensitive to the threshold used to differentiate low and high alpha state, such that significant correlations were present between alpha state parameter and behavioral outcome only when threshold for high alpha state was set as above 30% of maximum alpha power during eyes-open resting-state and not for the higher thresholds. There was a marginally significant positive trend between change in pain ratings and mean alpha power, however, the direction of this trend was opposite to expected.

Being able to measure any therapeutic change sensitively and displaying this as feedback to the patients is important in order to reinforce mental strategies which lead to an increase in alpha. Failure to detect such small changes may prevent patients from recognizing practices which lead to these changes as the required positive or negative feedback would not be provided. Our results have a number of implications in terms of designing future neurofeedback studies. Firstly, it has implications in terms of EEG data analysis that should be performed in neurofeedback studies, whereby we encourage other researchers to not only report changes in mean alpha amplitude but also to report changes in parameters of alpha states dynamics. This will enable us to assess whether a patient is actually responding to neurofeedback therapy and also allow us to further our understanding of how the brain responds to such therapy. Secondly, such dynamic alpha states parameters can also be used to fine-tune the way neurofeedback is delivered. Feedback signals shown to the patients can be based on such alpha dynamic features rather than a cruder measure such as mean amplitude. Considering that these parameters are more reflective of the bistable dynamics of alpha rhythm, providing feedback based on them may lead to a more physiologically meaningful (causal) change in alpha power and encourage control strategies which actually translate to pain reduction.

These results suggest that perhaps changes in fractional occupancy, mean dwell times and transition probability may have different implications in terms of the functioning of the underlying neuronal network than changes in mean amplitude alone, and it might be the case it is changes in these dynamic parameters which might be responsible for the increased resilience to pain. For instance, it has been suggested that longer dwell times might reflect an increased stability of the network giving rise to the respective alpha state (Khanna et al., 2015). Increased transition probability means an increased chance of sequential activation of the state (Khanna et al., 2015). Pain relief may be mediated through increased transitioning of individuals’ alpha from low to high alpha state as it may reflect the receptiveness of the brain for the incoming stimulus (Michel and Koenig, 2018). Another reason why such patterns of sequential activation might influence pain might be that the sequence in which these alpha states are present may have a meaning in itself and influence how the incoming stimulus is being processed (Buzsáki and Watson, 2012; Khanna et al., 2015; Michel and Koenig, 2018). This has been suggested before based on the neuronal workspace model where alpha states have been interpreted as “atoms of thoughts” (Ros et al., 2014; Villafaina et al., 2019), each representing specific mental processes making up a conscious mind. Hence, the sequence of mental processes or transition properties from one state to another can be thought to carry a meaning in itself (Ros et al., 2014; Villafaina et al., 2019).

Another interesting observation was regarding the presence of changes in alpha state parameters in the chronic pain patients with little consistent change in healthy volunteers. One possible explanation for this might be that chronic pain patients have more scope for improvement in these parameters since their alpha state parameters were lower, although not significantly, than healthy participants during the first session. However, another explanation for this might be that perhaps neurons are more susceptible to plasticity by external factors in chronic pain patients than healthy individuals. This idea of chronic pain patients having lower alpha than healthy participants and lower alpha being correlated with greater pain has been reported widely in the literature (Jensen et al., 2013b; Lim et al., 2016; Villafaina et al., 2019).

This discrepancy in the EEG changes between chronic pain patients and healthy participants may also be due to a motivational effect. We did not assess the motivation of our participants at the beginning of the training, hence the impact of any such factor on the performance of the individual cannot be determined in our study. Nevertheless, our results show that chronic pain patients are as capable, if not more capable, than healthy participants at learning to control their alpha activity. One would have anticipated that since the neuronal networks have already been rewired in chronic pain patients to attend to pain signals (Katz and Rothenberg, 2005; Reddan and Wager, 2019), it might be more difficult for chronic pain patients to change their brain activity following prolonged exposure to pain. Our results suggest that brains of chronic pain patients might be more plastic than we think. If this is the case, then such neuromodulatory therapy may have substantial potential for systematic development for personalized therapy in the future.

The initial rationale for alpha states modeling in this paper was simply to have a description of the dynamics of alpha power fluctuations between the low and high alpha states. Since the participants were required to follow the feedback provided to them, an extrinsic threshold, used to define these two “empirical” states, was derived based on an *ad hoc* criterion of what alpha power would be considered “good” or “high” enough for the neurofeedback to have an effect. There are no suggestions in the literature in terms of what this threshold should be, hence, we analyzed our data for a range of thresholds.

Ideally however, this threshold can be determined based on a characterization of the bistable dynamics of the endogenous alpha fluctuations of each subject, rather than based on an external criterion. The bistable nature of the alpha rhythm means that whilst in the low alpha state, the amplitude of the alpha fluctuates around a low “mean” value for some time and then spontaneously switches to the high alpha state, where it starts fluctuating around a high “mean” alpha value (Freyer et al., 2009, 2011; Roberts et al., 2015). This gives a bimodal distribution of the alpha amplitudes when viewed in double logarithmic coordinates, which can be best described by the sum of two exponential distributions (one for each alpha mode) (Freyer et al., 2009; Roberts et al., 2015). Hence the “meeting point” of the two distributions, (which is not the same as the midpoint of the low and high mean values), would be a more physiologically meaningful threshold for defining the two intrinsic alpha states. Moreover, changes in such an “intrinsic” threshold evaluated pre and post neurofeedback intervention, might correlate with a pain effect, which might in itself be a potential marker of pain modulation. Hence, although the extrinsic threshold used in our study might be sub-optimal, these preliminary findings provide useful insights into the underlying mechanisms of neurofeedback which warrant further in-depth analysis.

The limiting factors of our study were the small sample size and the small number of sessions. This means the results should be interpreted with caution. This could perhaps explain why we did not see a significant increase in the different EEG parameters. It might be the case that with provision of further NFB sessions, we would have seen significant increase. However, this preliminary study has enabled us to identify learning indices which can still detect learning in these initial sessions when the other commonly used parameters do not show noticeable change. In fact, the alpha states dynamic approach presented here might be a better way to model alpha rhythm as it exploits the bistable nature of alpha rhythm better.

On a methodical note, we used a sliding window to calculate the alpha power. This approach introduces a smoothing effect that can “smear out” the finer dynamics of the alpha rhythm. Since the alpha rhythm shows some scale invariance (Van De Ville et al., 2010), this short window averaging will still preserve some of the dynamical features of the alpha rhythm, which we believe support results reported here. To improve on this, we propose that future analysis can then focus on characterizing the dynamics of the instantaneous alpha power fluctuations. Such an approach has the potential to uncover new and possibly more sensitive effects of α-NFB for the treatment of chronic pain.

## Conclusion

In conclusion, we have observed changes in dynamic alpha state parameters that are not reflected in mean alpha power during alpha neurofeedback for pain. Our study found that changes in alpha state parameters might potentially be more sensitive predictors of learning than currently used measures. Over the course of five alpha neurofeedback sessions in this study, whilst there was no change in mean alpha power, there was a trend of increase in fractional occupancy, dwell time duration, and transition probability of high alpha state. Furthermore, fractional occupancy, mean dwell times and transitional probability was correlated with change in pain scores, such that in sessions where an individual spends more time in the high alpha state, had longer high alpha state visits or had higher probability of transitioning from low to high alpha state were likely to report greater reduction in pain. We hope that our results will encourage others to measure learning using similar approach. Reporting such temporal dynamics alongside changes in mean alpha power will not only enable us to measure success more sensitively but may also provide insight into the mechanisms of neurofeedback training.

## Data Availability Statement

The raw data supporting the conclusions of this article will be made available by the authors, without undue reservation, to any qualified researcher.

## Ethics Statement

The studies involving human participants were reviewed and approved by the National NHS Research Ethics Committee. The patients/participants provided their written informed consent to participate in this study.

## Author Contributions

KP, JH, HS, JT, AC, KL-D, CB, AJ, MS, and NT-B designed the study. KP, JH, and HS conducted the study, including patient recruitment, data collection, and data analysis. KP prepared the manuscript draft with important intellectual input from JH, HS, JT, AC, KL-D, CB, AJ, MS, and NT-B during interpretation of the data and refining of the manuscript. All authors approved the final manuscript.

## Conflict of Interest

The authors declare that the research was conducted in the absence of any commercial or financial relationships that could be construed as a potential conflict of interest.
