# Supplementary figures and images for: Using EEG Alpha States to Understand Learning During Alpha Neurofeedback Training for Chronic Pain
Source: Front Neurosci. 2021 Feb 22;14:620666. doi: 10.3389/fnins.2020.620666 (PMC7958977; doi:10.3389/fnins.2020.620666)

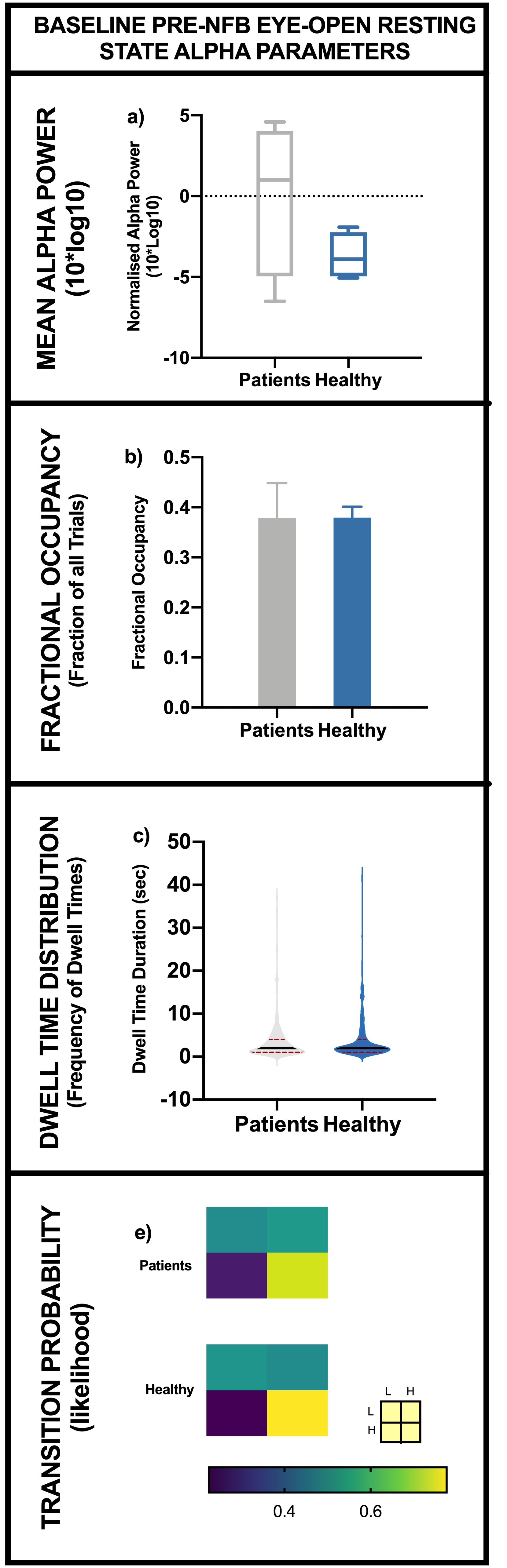

Supplement: Supplementary file 1 [file Image_1.JPEG]
